# A rapid review of differences in cerebrospinal neurofilament light levels in clinical subtypes of progressive multiple sclerosis

**DOI:** 10.3389/fneur.2024.1382468

**Published:** 2024-04-09

**Authors:** Haritha L. Desu, Katherine M. Sawicka, Emily Wuerch, Vanessa Kitchin, Jacqueline A. Quandt

**Affiliations:** ^1^Neuroimmunology Unit, Centre de Recherche du Centre Hospitalier de l’Université de Montréal (CRCHUM), Montreal, QC, Canada; ^2^Department of Neurosciences, Université de Montréal, Montreal, QC, Canada; ^3^Child Health Evaluative Sciences Program, Research Institute, The Hospital for Sick Children, Toronto, ON, Canada; ^4^Department of Medicine, Division of Neurology, University of Toronto, Toronto, ON, Canada; ^5^Institute of Health Policy, Management and Evaluation, Dalla Lana School of Public Health, University of Toronto, Toronto, ON, Canada; ^6^Hotchkiss Brain Institute and the Department of Clinical Neuroscience, University of Calgary, Calgary, AB, Canada; ^7^University of British Columbia Library, Vancouver, BC, Canada; ^8^Department of Pathology and Laboratory Medicine, University of British Columbia, Vancouver, BC, Canada; ^9^Djavad Mowafaghian Centre for Brain Health, University of British Columbia, Vancouver, BC, Canada

**Keywords:** multiple sclerosis, biomarkers, progression, neurofilament light, neurodegeneration

## Abstract

**Background:**

Multiple sclerosis (MS) is divided into three clinical phenotypes: relapsing–remitting MS (RRMS), secondary progressive MS (SPMS), and primary progressive MS (PPMS). It is unknown to what extent SPMS and PPMS pathophysiology share inflammatory or neurodegenerative pathological processes. Cerebrospinal (CSF) neurofilament light (NfL) has been broadly studied in different MS phenotypes and is a candidate biomarker for comparing MS subtypes.

**Research question:**

Are CSF NfL levels different among clinical subtypes of progressive MS?

**Methods:**

A search strategy identifying original research investigating fluid neurodegenerative biomarkers in progressive forms of MS between 2010 and 2022 was applied to Medline. Identified articles underwent title and abstract screen and full text review against pre-specified criteria. Data abstraction was limited to studies that measured NfL levels in the CSF. Reported statistical comparisons of NfL levels between clinical phenotypes were abstracted qualitatively.

**Results:**

18 studies that focused on investigating direct comparisons of CSF NfL from people with MS were included in the final report. We found NfL levels were typically reported to be higher in relapsing and progressive MS compared to healthy controls. Notably, higher NfL levels were not clearly associated with progressive MS subtypes when compared to relapsing MS, and there was no observed difference in NfL levels between PPMS and SPMS in articles that separately assessed these phenotypes.

**Conclusion:**

CSF NfL levels distinguish individuals with MS from healthy controls but do not differentiate MS subtypes. Broad biological phenotyping is needed to overcome limitations of current clinical phenotyping and improve biomarker translatability to decision-making in the clinic.

## 1 Introduction

Multiple sclerosis (MS) is currently categorized using three clinical phenotype descriptions – relapsing–remitting MS (RRMS), primary progressive MS (PPMS), and secondary progressive MS (SPMS) (1). These categories have largely informed the design and implementation of research studies and treatment strategies in MS to date. However, major limitations in the use of clinical phenotyping in MS remain: significant heterogeneity of clinical features of MS, overlap in the biology associated with the pathophysiology of relapsing and progressive MS, and inconsistent designations of clinical disease activity. There has been a recent push to re-evaluate the conventional clinical course descriptors and consider instead some integration of radiological and biological phenotyping (2, 3) to improve our understanding of disease onset, treatment response and progression.

Until relatively recently, biological phenotyping has predominantly focused on studying fluid biomarkers that reflect otherwise “early” inflammatory or “later” neurodegenerative processes. With the recognition of shared biology between progressive and relapsing MS, there is increasing appreciation that neurodegenerative processes once associated with progressive disease are present throughout the “life of MS,” and each warrant investigation for their potential as targets to lessen disability or improve repair (4, 5). A number of reviews have recently focused on the mechanisms thought to drive progression in MS, ranging from inflammation originating in the periphery to that sustained by lymphoid or myeloid populations within the central nervous system (CNS), oxidative stress and associated energy imbalance/mitochondrial dysfunction, neuronal and axonal injury through direct and indirect mechanisms, and decreased potential to remyelinate or repair damage (6, 7).

Neuronal dysfunction and degeneration are central events in Alzheimer’s disease, Parkinson’s disease, amyotrophic lateral sclerosis and MS. In MS, neuronal dysfunction may be linked to cell death, synaptic loss, and disrupted neuronal cell signaling which also may follow primary or secondary demyelinating events; each of these can occur in the context of inflammation to differing degrees early or late in the disease (8–11). Neurodegeneration from this wide range of insults, measured as CNS atrophy, has been documented in RRMS and SPMS in both white matter (WM) and gray matter (GM) (12, 13). The clearest distinctions between the pathology of progressive vs. RRMS have been grounded in histological evaluations. Over the past two decades, immunohistochemical studies have shown that compared to the predominant WM damage in RRMS, GM involvement is far more extensive in progressive MS than in RRMS (14). These studies focused not only on the extent and location of demyelination, but also specific inflammatory aggregates unique to the meninges and other GM regions in addition to the limited and incomplete remyelination more often observed in progressive MS (15–17). Connecting these histological observations in biopsy or autopsy specimens to MS pathophysiology and clinical changes in patients highlights the need to identify a combination of accessible biomarkers to inform on disease processes both temporally and anatomically. There remains a gap in understanding the relevance and suitability of neurodegenerative fluid biomarkers in distinguishing patterns of progressive disease, namely secondary progressive and primary progressive subtypes, to better understand progression.

Neurofilament light chain (NfL) is a fluid biomarker of particular interest when it comes to phenotyping MS. NfL is the smallest subunit of the neuronally-restricted family of neurofilaments constituting neuronal and axonal cytoskeleton in the nervous system. Detection of NfL in blood or cerebrospinal fluid (CSF) reflects primary neurodegenerative or secondary neurodegenerative injury due to trauma or inflammation in the CNS (18). In the context of MS, CSF NfL has been proposed as a reliable biomarker for diagnosis and disease monitoring (19–21). In a recent meta-analysis, CSF NfL was shown to be higher in MS subjects compared to controls and inconsistently able to differentiate between subjects with RRMS and progressive MS (22). It is unknown whether CSF NfL can reliably differentiate between SPMS and PPMS.

In this study we conduct a rapid review to (1) examine the landscape of neurodegenerative fluid biomarkers in the context of progressive forms of MS and (2) qualitatively describe relative measures of a proposed marker of neurodegeneration and progression, CSF NfL, in progressive forms of MS to determine if CSF NfL would be useful in differentiating clinical phenotypes of progressive MS.

## 2 Methods and results

### 2.1 Design and conduct of rapid review

This rapid review was informed by the development protocol for the upcoming Preferred Reporting Items for Systematic Reviews and Meta-Analyses (PRISMA) rapid review guidelines (23) and in accordance with the Interim Guidance from the Cochrane Rapid Reviews Methods Group (24). Rapid review methodology was selected as its methods were best suited to the narrow focus and time frame available for this study.

A search strategy (Supplementary File 1) of the Ovid MEDLINE database was created in collaboration with a medical research librarian (VK). A combination of keywords and medical subject headings (MeSH) were applied to each concept (Multiple Sclerosis and fluid neurodegenerative biomarkers). The search was modified by removing irrelevant evidence synthesis types (systematic review and meta-analyses) as well as case reports in order to refine results to primary research. The search results were further limited to published and indexed articles between 1 January 2010 and 19 December 2022 and available in the English language. Our intent was to focus on publications most likely to employ the most recent disease classification, utilizing CIS, RRMS, SPMS and PPMS, while also capturing the time frame when MS disease modifiers distinguishing active from inactive disease were introduced (1).

Three review authors (HD, KS, EW) performed title/abstract screening using the Covidence platform. Each study required two votes to pass through to full-text review. Any discrepancies were vetted by a subject expert (JQ). At the full-text review stage, the potentially eligible studies were reviewed in full by one review author (HD, KS, or EW) and were checked by a second reviewer if the first reviewer was unsure of inclusion.

Of 8,511 records identified, 15 duplicates were identified and removed using Covidence, 8,496 were screened at the title/abstract level, and 440 full-text studies were assessed for eligibility. The criteria for biomarker eligibility included a focus on glial, myelin or neuroaxonal components/proteins enriched within the nervous system (brain or spinal cord) per the Human Protein Atlas at proteinatlas.org (25). Biomarkers reflected components expressed and thus potentially “freed” from the CNS into the fluid (CSF, serum or plasma) as a secondary result of damage. They could be proteins or enzymes; however, lipids, hormones/metabolites and amino acids as well as antibodies to any of the above were excluded. Of the 440 full-text studies screened, 147 studies were included for abstraction. One reviewer (HD, KS or EW) abstracted each of the 147 studies for the fluid type and biomarker measured in the studies. The focused abstraction of the studies that compared NfL levels in the CSF between clinical phenotypes was completed by two independent reviewers per study (HD, KS, and/or EW).

### 2.2 Data abstraction and synthesis

**Figure 1:**
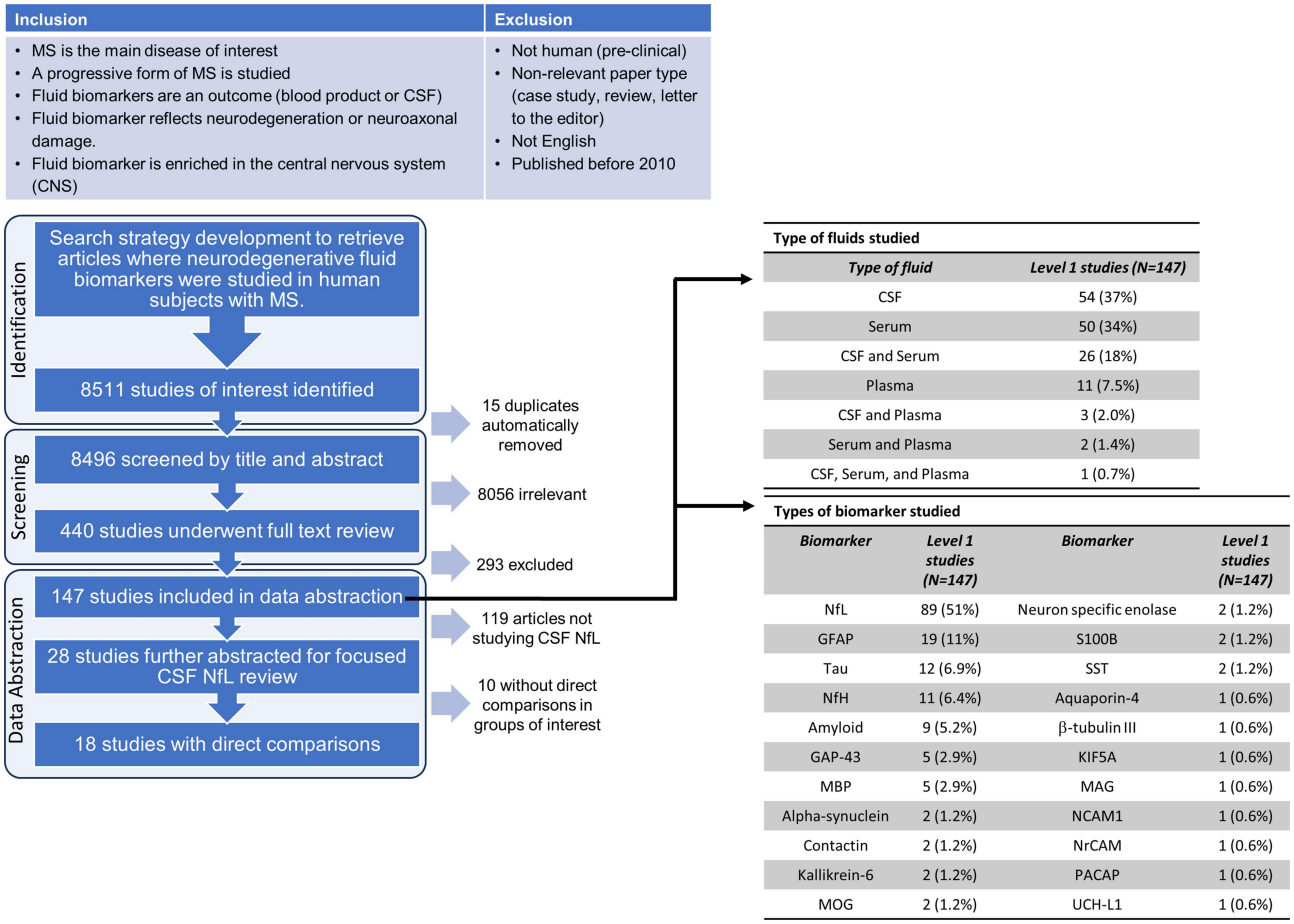
Flow chart of the inclusion/exclusion criteria, screening and selection process for the rapid review. Abbreviations for the biomarkers evaluated in the abstracted data studies: CSF, cerebrospinal fluid; NfL, neurofilament light; GFAP, glial fibrillary acidic protein; NfH, neurofilament heavy; GAP-43, growth associated protein 43; MBP, myelin basic protein; MOG, myelin oligodendrocyte glycoprotein; SST, somatostatin; KIF5A, kinesin family member 5A; MAG, myelin associated glycoprotein; NCAM1, neural cell adhesion molecule 1; NrCAM, neuronal cell adhesion molecule; PACAP, pituitary adenylate-cyclase-activating polypeptide; UCH-L1, Ubiquitin C-terminal hydrolase L1.

To determine the general landscape since 2010 of what type of neuroaxonal/degenerative biomarkers and what types of fluids were measured/studied, we extracted this information from the 147 studies that originally met our inclusion criteria during the full-text review. Out of the 147 studies, approximately one-third measured CSF alone, one-third in serum alone, and the final third consisted of studies that measured biomarkers in plasma or a combination of the three (Figure 1). By far, most of the abstracted studies measured NfL in the fluid (89 studies, 51%, Figure 1). The second most frequently measured biomarker was GFAP (19 studies, 11%). The remaining biomarkers we abstracted from the studies were measured in a total of 1–12 studies. Since NfL was the most frequently measured biomarker in our focus area in the last 13 years and CSF was the most frequently used fluid type in the studies, we decided to focus this rapid review on qualitatively assessing whether NfL levels in the CSF were found to be different in the progressive MS phenotypes in comparison to HC and RRMS. Using this criteria, 28 studies were identified to measure NfL in the CSF.

To abstract only results that could be qualitatively assessed in relation to other studies, 10 out of the 28 studies that measured NfL levels in the CSF were excluded as they did not report on direct statistical comparisons of means or medians. The remaining 18 (26–43) studies were abstracted for the type of statistical test, type of progressive MS studied, statistical comparisons made, and reported differences in NfL (higher, lower, or not significant). Of those 18 studies, 10 studies conducted a t-test or equivalent statistical test, 7 studies conducted an ANOVA, and 2 conducted an ANCOVA. All studies that applied an ANOVA test also compared individual groups employing a post-hoc multiple comparison’s test. One study by Sellebjerg et al. (41), compared their patient cohorts to healthy controls using an ANOVA with multiple comparisons and then compared between their patient populations using a t-test (or equivalent).

Furthermore, out of the 18 studies, six studies pooled progressive MS subtypes, four studies did not define their progressive MS population, five studies separately assessed PPMS and SPMS, two studies assessed PPMS only and one study only included an SPMS patient cohort. Overall, there was no clear pattern or consistency in grouping and/or separation of MS phenotypes.

### 2.3 CSF NfL levels rarely elevated in progressive over relapsing-remitting MS subtypes

**Figure 2:**
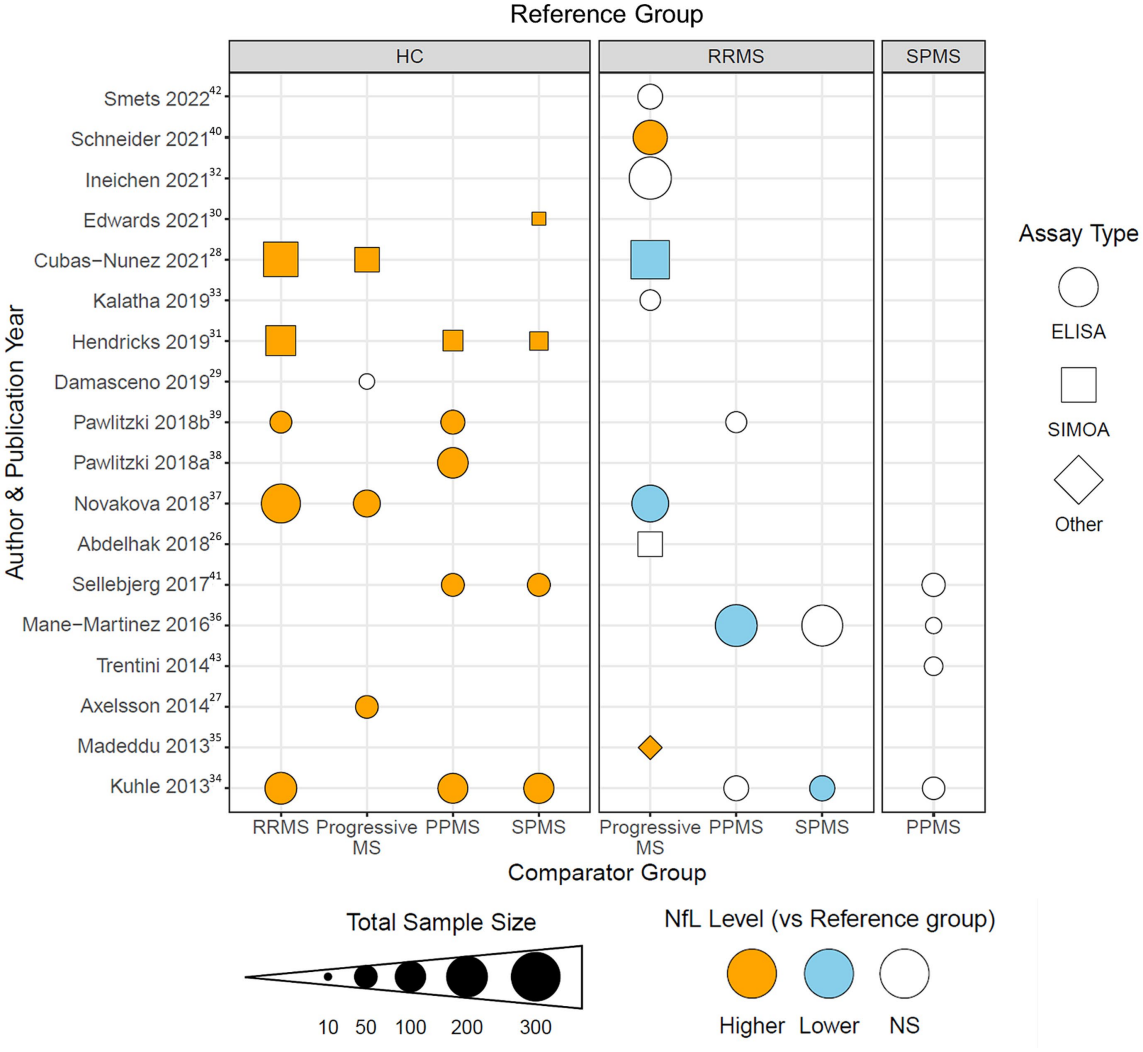
Dot plot visualization demonstrating the reported differences in CSF NfL in MS subtypes for each of the 18 studies included in the level 2 analysis. The reference clinical subtype is listed in the gray boxes at the top of the figure. The comparison clinical subtype is listed at the bottom of the figure. Each shape is color coded for if CSF NfL levels were reported to be higher (orange), lower (blue), or non-significantly different (white) in the comparison group compared to the reference group for the study listed on the same horizonal axis. Dot sizes correspond to total sample size for the abstracted study. Shape corresponds to type of assay used in the study: enzyme-linked immunosorbent assay (ELISA, circle), single-molecule array (Simoa, triangle), and other (diamond). Superscripts in the figure correspond to citations listed in the bibliography.

To visualize the reported differences identified with our data abstraction, a dot plot was generated using R statistical software version 4.2.1 (44) and the ggplot2 package (45) (Figure 2). 9 out of the 10 studies that directly compared any MS phenotype to healthy controls reported higher NfL levels in the MS phenotypes. In contrast, in 11 studies that compared NfL levels in progressive MS phenotypes to RRMS, the trends were quite different irrespective of whether the progressive population was defined, pooled, or separated into PPMS and SPMS. In the eight studies that either did not define or pooled the progressive MS cohorts into one group, two found NfL to be higher than RRMS, two found NfL to be lower and 4 found NfL to be comparable. Of the three studies that compared CSF NfL in PPMS to RRMS, two smaller studies showed the levels to be comparable and a third by Mane-Martinez et al., detected lower levels of NfL in people with PPMS than RRMS. Similarly, the two studies comparing SPMS to RRMS differed with one showing comparable levels in each and the other detecting lower NfL in SPMS than RRMS. Only four of the 18 studies directly compared CSF NfL levels between PPMS and SPMS and none identified differences (Figure 2). Furthermore, the type of assay used to measure CSF NfL was not a differentiating factor for the mixed results across the studies that compared progressive MS types to RRMS (Figure 2).

Although disease activity assessments were introduced as modifiers in the MS clinical phenotyping criteria (1), only 9 out of the 18 studies assessed disease activity in participants using clinical (typically relapse) and/or radiological features (typically gadolinium (Gd)-enhancing lesions). In Cubas-Nunez et al. (28), activity was assessed only in the pooled RRMS group and CSF NfL levels were greater in active MS than in remitting MS participants. Activity in the progressive groups was only assessed in 2 of the 11 studies that compared progressive to RRMS groups. In both studies, the progressive MS groups were pooled (PMS) and distinguished as active vs. inactive PMS, and in each study, active PMS participants had significantly higher CSF NfL levels than inactive PMS participants (27, 41). Differences attributed to activity were not shown in Figure 2 because we could not determine whether activity made any difference or distinction in those comparisons.

## 3 Discussion

The pathology underlying RRMS has become increasingly well understood, facilitating the identification of candidate biomarkers (46–48). In contrast, there is a gap in knowledge surrounding similarities or differences in SPMS vs. PPMS pathology, reflected by a lack of biomarkers to differentiate them from RRMS or each other. While diagnoses of RRMS, PPMS and SPMS are typically done based on clinical presentation (i.e., presence/absence of relapses, clinical worsening), questions remain regarding whether these discrete clinical definitions accurately represent underlying pathology. The alternative would involve a bypass of classification entirely, and disease would instead be viewed along a spectrum with a focus on biological phenotyping and a more mechanism-based framework to understand and characterize disease onset and progression (3, 49–51). To date, MRI has been one of the most readily employed tools to assess disease activity and damage as well as monitor response to therapy in individuals living with MS (52), yet standard imaging approaches may not distinguish the pathobiology of MS with appreciable specificity compared to the sensitivity it affords (53). Thus, there is increasing interest in employing more advanced imaging approaches (54, 55) along with biomarkers as biological readouts to increase our understanding of processes driving damage and potentially also, repair.

Progression is characterized clinically by the accumulation of neurological disability without recovery, yet our appreciation of when progression begins has changed markedly. Accumulation of disability in MS may occur as relapse-associated worsening (RAW) or steady progression independent of relapse activity (PIRA), with PIRA historically regarded as a feature of primary and secondary progressive MS. Indeed, PIRA has been demonstrated early in MS, both in CIS and RRMS (56). Seminal studies examining large cohorts have evaluated markers linked to neurodegeneration, and in the case of NfL, found it elevated 1 year before and in some cases as early as 6 years before the first MS symptom (57).

In the present study, we compared CSF NfL levels across MS subtypes and evaluated the potential of CSF NfL to discern PPMS from SPMS. While NfL levels were consistently elevated in participants with MS compared to healthy controls, CSF NfL was more often comparable to or lower in progressive MS compared to RRMS.

These findings are consistent with previous studies, where NfL levels were shown to be higher in MS vs. healthy controls (58, 59), but not significantly different between MS subtypes (60). Although NfL has been proposed as a marker of progressive MS (61), our findings suggest NfL is not a suitable biomarker of progression in relation to the distinction of progressive from relapsing MS subtypes. While one previous systematic review identified elevated NfL in progressive vs. RRMS, these changes were observed in the periphery, not CSF (19). Because we focused on CSF, we do not know whether progressive vs. relapsing or more specifically, progressive subtype differences, may be detectable in the periphery (i.e., plasma or serum). While evaluation of CSF remains perhaps the most robust measure of optic nerve or CNS-specific degeneration, limiting our abstracted studies to CSF only and to those specifically reporting direct statistical comparisons between MS subtypes clearly reduced the number and types of studies included in our assessment compared to systematic reviews.

Disease activity has become an important consideration when evaluating MS disease, and also in evaluating response to therapy. Indeed, the association of disease activity with drug efficacy in SPMS patients has led to recent FDA approvals of several drugs specifically for treatment of *active* secondary progressive disease, including siponimod, cladribine, ofatumumab and ponesimod (62–65). We found that half the studies we examined did not include information on the disease activity of participants, and only a handful grouped participants based on observed activity. Notably, when activity *was* evaluated, NfL levels were higher in active vs. inactive progressive patients (27, 41). In contrast, in studies that reported significant differences in NfL levels between progressive and RRMS patients, it is unclear whether these findings could be explained by the activity of the progressive group, as this was not directly compared. However, one possible explanation for the progressive populations reviewed typically having lower NfL than the RRMS populations may be explained by the inclusion of progressive participants that had relatively inactive MS overall: the proportion of active PMS participants was typically less than one-third and often less than one-tenth. The similarities in cNFL levels in RRMS and in relatively inactive PMS individuals in studies examined in this review are consistent with other studies showing NfL correlations with radiological or clinical measurements of activity; together these findings propose NfL is a marker of disease activity rather than progression (66).

Disease duration (DD) has been linked to progressive subtypes, however DD at any subgroup level was only provided in 12/18 studies which precluded our evaluating its association with NfL levels. A comparison of mean DD duration between the studies in our review showed subgroup differences in DD: 57 months for RRMS patients vs. 161 months in PMS, and 199 months in SPMS vs. 89 months in PPMS. These values are similar to a meta-analysis of CSF NfL across MS subgroups, where DD in SPMS was typically double that of PPMS yet also showed no difference between PPMS and SPMS participants (67). Indeed, studies showing no correlation of DD with CSF NfL in PPMS (38) and CSF NfL levels correlating negatively with DD as long as 30 years in large PMS studies (68) instead support NfL being reflective of inflammatory and/or neuroaxonal damage occurring in earlier stages of MS.

While some studies we examined directly compared PPMS vs. SPMS, others lumped all progressive participants together or did not specify clinical subtypes, making comparisons challenging. These grouping patterns were likely done to increase sample sizes and could also be explained by a lack of consensus regarding how best to distinguish progressive participants with MS in clinical studies. A limitation of our study to examine differences in neurodegenerative markers in the two progressive forms of MS lies in the potential bias of investigators to group these subtypes under an assumption that neurodegeneration likely exists in both groups, as neurodegeneration has largely been equated to the progressive and typically, later, stage of the disease. The majority of studies (13 out of 18) used an enzyme-linked immunosorbent assay (ELISA) to measure CSF NfL. These ELISA assays had a 30 pg./mL lower limit of detection that, while suitable for CSF, is not recommended for serum where NfL levels are typically 50–100 times lower than in the CSF (34). It is plausible studies employing higher sensitivity platforms such as single-molecular array or Simoa (69) might better detect differences among MS subgroups, yet this is not requisite given mean CSF NfL levels in MS studies typically range from 800 to 2,200 pg./mL vs. 250–550 pg./mL in controls (67). While one study using Simoa did not identify any differences between PMS and RRMS participants, the second did show CSF NfL was lower in PMS than in RRMS groups (26, 28). Notably, a meta-analysis showing a higher CSF NfL in RRMS over progressive MS participants (with a small, albeit significant effect size) utilized the same ELISA employed in 12 of the 13 ELISA studies in our review (67).

The promise of a single biomarker such as NfL to inform on disease progression has already lost luster, and the reasoning may ultimately have roots in how researchers and clinicians define *biological* progression. NfL levels may reflect specific pathology involving active axonal damage/degeneration early in the disease process well, but interpretation of higher levels are confounded later by comorbidities such as aging or obesity, consistent with the sensitivity of NfL for neuroaxonal damage but little specificity for any one MS-specific biological phenomenon (66). Even in early MS, cases where serum NfL values are high in otherwise stable patients serve as reminders of the need to consider alternative causes for the high levels and comorbidities including trauma, CNS microvascular disease or polyneuropathies need to be considered.

Moving forward, future studies should evaluate the levels of a broader panel of degenerative biomarkers in people with MS and importantly, include assessments longitudinally from onset through worsening. To this point, biomarkers of neuroaxonal and glial damage/dysfunction such as tau or GFAP, have been linked to progression more specifically than NfL (70–72) and would provide clarity on whether late disease or perhaps “later life” progression is classified currently as SPMS and PPMS largely share pathological processes or, alternatively, might represent completely distinct manifestations. Biomarker comparisons between clinical phenotypes have yielded interesting clues where radiological (73) (extent of Gd lesions or focal WM lesion load), histological (74) (extent of meningeal infiltrates or extent of white matter lesion) and inflammation-associated markers (75, 76) [CD5L or chitinase-3-like protein 1 (CHI3L1)], as well as select metabolites (77) (ascorbate), were found to differ between SPMS and PPMS. Such studies highlighted inflammation-related differences between progressive phenotypes, and careful interpretation following their evaluation regularly over early and later intervals may discern their potential as key pathological hallmarks rather than epiphenomena. Future studies, whether more foundational or clinical, should aim to include descriptions of clinical and/or radiological disease activity whenever possible, to allow for a better understanding of the relevance of different biomarkers to specific disease processes in people over their lifetime with MS. A focus on biological phenotyping should replace the current clinical subtyping in making predictions or drawing conclusions on treatment and prognosis in MS.

## Author contributions

HD: Data curation, Investigation, Writing – original draft, Writing – review & editing, Formal analysis, Visualization. KS: Data curation, Investigation, Writing – original draft, Writing – review & editing, Formal analysis, Visualization. EW: Data curation, Writing – original draft, Writing – review & editing, Formal analysis, Visualization. VK: Conceptualization, Data curation, Methodology, Writing – original draft, Writing – review & editing, Software. JQ: Conceptualization, Data curation, Investigation, Methodology, Supervision, Validation, Writing – original draft, Writing – review & editing.
