# Supplementary material for: A rapid review of differences in cerebrospinal neurofilament light levels in clinical subtypes of progressive multiple sclerosis
Source: Front Neurol. 2024 Apr 9;15:1382468. doi: 10.3389/fneur.2024.1382468 (PMC11035744; doi:10.3389/fneur.2024.1382468)
Supplement: Supplementary file 1 [file Data_Sheet_1.DOCX]

Supplementary File 1: Ovid MEDLINE Search Strategy

**Database: Ovid MEDLINE(R) and Epub Ahead of Print, In-Process, In-Data-Review & Other Non-Indexed Citations, Daily and Versions <1946 to Dec 19, 2022>**

**Search Strategy:**

| **Search Line #** | **Search Query** | **Results** |
| --- | --- | --- |
| 1 | exp Multiple sclerosis/ | 68374 |
| 2 | Demyelinating diseases/ | 12788 |
| 3 | Myelitis, Transverse/ | 1683 |
| 4 | Optic neuritis/ | 6431 |
| 5 | (multiple sclerosis or optic neuritis or transverse myelitis or clinically isolated syndrome or demyelinating or balo*).tw,kf. | 108142 |
| 6 | or/1-5 | 123782 |
| 7 | Biomarkers/ | 340922 |
| 8 | biomarker*.tw,kf. | 383739 |
| 9 | Cerebrospinal Fluid/ | 204462 |
| 10 | cerebrospinal fluid.tw,kf | 99918 |
| 11 | ((axon* or biologic or clinical or laboratory or serum) adj1 marker*).tw,kf. | 18969 |
| 12 | neuroaxonal.tw,kf. | 1039 |
| 13 | Intermediate Filaments/ | 4545 |
| 14 | neurofilament*.tw,kf. | 13615 |
| 15 | Myelin Sheath/ | 19040 |
| 16 | myelin.tw,kf. | 41938 |
| 17 | neurodegener*.tw,kf. | 143727 |
| 18 | or/7-17 | 902720 |
| 19 | review.pt. | 3074530 |
| 20 | meta-analysis/ or systematic review/ or systematic reviews as topic/ or meta-analysis as topic/ or "meta analysis (topic)"/ or "systematic review (topic)"/ or exp technology assessment, biomedical/ or network meta-analysis/ | 333537 |
| 21 | (meta-analy* or metaanaly*).ti. | 167928 |
| 22 | ((systematic or umbrella or scoping or critical or literature or rapid or mixed or mapping) adj1 review*).ti. | 282566 |
| 23 | Case Reports/ | 2311916 |
| 24 | (case$ adj2 (stud* or series)).tw,kf. | 393061 |
| 25 | or/19-23 | 5473571 |
| 26 | 6 and 18 | 28759 |
| 27 | 26 not 25 | 20289 |
| 28 | 27 not (exp animals/ not humans.sh.) | 14632 |
| 29 | limit 28 to (english language and yr="2010 -Current") | 8511 |
