# Supplementary material for: A rapid review of differences in cerebrospinal neurofilament light levels in clinical subtypes of progressive multiple sclerosis
Source: Front Neurol. 2024 Apr 9;15:1382468. doi: 10.3389/fneur.2024.1382468 (PMC11035744; doi:10.3389/fneur.2024.1382468)
Supplement: Supplementary file 2 [file Data_Sheet_2.DOCX]

Supplementary File 2

Studies examined in Data Abstraction

(1-147)

1. Abdelhak A, Huss A, Kassubek J, Tumani H, Otto M. Serum GFAP as a biomarker for disease severity in multiple sclerosis. Scientific reports. 2018;8(1):14798.

2. Aeinehband S, Lindblom RPF, Al Nimer F, Vijayaraghavan S, Sandholm K, Khademi M, et al. Complement component C3 and butyrylcholinesterase activity are associated with neurodegeneration and clinical disability in multiple sclerosis. PloS one. 2015;10(4):e0122048.

3. Al-Keilani MS, Almomani BA, Al-Sawalha NA, Al Qawasmeh M, Jaradat SA. Significance of serum VIP and PACAP in multiple sclerosis: an exploratory case-control study. Neurological sciences : official journal of the Italian Neurological Society and of the Italian Society of Clinical Neurophysiology. 2022;43(4):2621-30.

4. Alcala C, Cubas L, Carratala S, Gascon F, Quintanilla-Bordas C, Gil-Perotin S, et al. NFL during acute spinal cord lesions in MS: a hurdle for the detection of inflammatory activity. Journal of neurology. 2022;269(7):3495-500.

5. Amor S, van der Star BJ, Bosca I, Raffel J, Gnanapavan S, Watchorn J, et al. Neurofilament light antibodies in serum reflect response to natalizumab treatment in multiple sclerosis. Multiple sclerosis (Houndmills, Basingstoke, England). 2014;20(10):1355-62.

6. Anderson V, Bentley E, Loveless S, Bianchi L, Harding KE, Wynford-Thomas RA, et al. Serum neurofilament-light concentration and real-world outcome in MS. Journal of the neurological sciences. 2020;417(jbj, 0375403):117079.

7. Antonelou RC, Emmanouilidou E, Gasparinatos G, Velona T, Voumvourakis KI, Stefanis L. Decreased levels of alpha-synuclein in cerebrospinal fluid of patients with clinically isolated syndrome and multiple sclerosis. Journal of neurochemistry. 2015;134(4):748-55.

8. Arslan B, Ayhan Arslan G, Tuncer A, Karabudak R, Sepici Dincel A. Evaluation of cerebrospinal fluid neurofilament light chain levels in multiple sclerosis and non-demyelinating diseases of the central nervous system: clinical and biochemical perspective. Bosnian journal of basic medical sciences. 2022;22(5):699-706.

9. Augutis K, Axelsson M, Portelius E, Brinkmalm G, Andreasson U, Gustavsson MK, et al. Cerebrospinal fluid biomarkers of beta-amyloid metabolism in multiple sclerosis. Multiple sclerosis (Houndmills, Basingstoke, England). 2013;19(5):543-52.

10. Avsar T, Korkmaz D, Tutuncu M, Demirci NO, Saip S, Kamasak M, et al. Protein biomarkers for multiple sclerosis: semi-quantitative analysis of cerebrospinal fluid candidate protein biomarkers in different forms of multiple sclerosis. Multiple sclerosis (Houndmills, Basingstoke, England). 2012;18(8):1081-91.

11. Axelsson M, Malmestrom C, Gunnarsson M, Zetterberg H, Sundstrom P, Lycke J, et al. Immunosuppressive therapy reduces axonal damage in progressive multiple sclerosis. Multiple sclerosis (Houndmills, Basingstoke, England). 2014;20(1):43-50.

12. Axelsson M, Malmestrom C, Nilsson S, Haghighi S, Rosengren L, Lycke J. Glial fibrillary acidic protein: a potential biomarker for progression in multiple sclerosis. Journal of neurology. 2011;258(5):882-8.

13. Ayoglu B, Haggmark A, Khademi M, Olsson T, Uhlen M, Schwenk JM, et al. Autoantibody profiling in multiple sclerosis using arrays of human protein fragments. Molecular & cellular proteomics : MCP. 2013;12(9):2657-72.

14. Ayrignac X, Le Bars E, Duflos C, Hirtz C, Maleska Maceski A, Carra-Dalliere C, et al. Serum GFAP in multiple sclerosis: correlation with disease type and MRI markers of disease severity. Scientific reports. 2020;10(1):10923.

15. Baldassari LE, Planchon SM, Bermel RA, Nakamura K, Fisher E, Feng J, et al. Serum neurofilament light chain concentration in a phase 1/2 trial of autologous mesenchymal stem cell transplantation. Multiple sclerosis journal - experimental, translational and clinical. 2019;5(4):2055217319887198.

16. Bartosik-Psujek H, Psujek M, Jaworski J, Stelmasiak Z. Total tau and S100b proteins in different types of multiple sclerosis and during immunosuppressive treatment with mitoxantrone. Acta neurologica Scandinavica. 2011;123(4):252-6.

17. Bartova R, Petrlenicova D, Oresanska K, Prochazkova L, Liska B, Turecky L, et al. Changes in levels of oxidative stress markers and some neuronal enzyme activities in cerebrospinal fluid of multiple sclerosis patients. Neuro endocrinology letters. 2016;37(2):102-6.

18. Benkert P, Meier S, Schaedelin S, Manouchehrinia A, Yaldizli O, Maceski A, et al. Serum neurofilament light chain for individual prognostication of disease activity in people with multiple sclerosis: a retrospective modelling and validation study. The Lancet Neurology. 2022;21(3):246-57.

19. Bhan A, Jacobsen C, Myhr KM, Dalen I, Lode K, Farbu E. Neurofilaments and 10-year follow-up in multiple sclerosis. Multiple sclerosis (Houndmills, Basingstoke, England). 2018;24(10):1301-7.

20. Bilge N, Simsek F, Yevgi R, Ceylan M, Askin S. Low serum Alpha-SYNUCLEIN and oligomer Alpha-SYNUCLEIN levels in multiple sclerosis patients. Journal of neuroimmunology. 2020;350(JA2, 8109498, hso):577432.

21. Bridel C, Verberk IMW, Heijst JJA, Killestein J, Teunissen CE. Variations in consecutive serum neurofilament light levels in healthy controls and multiple sclerosis patients. Multiple sclerosis and related disorders. 2021;47(101580247):102666.

22. Brune S, Hogestol EA, de Rodez Benavent SA, Berg-Hansen P, Beyer MK, Leikfoss IS, et al. Serum neurofilament light chain concentration predicts disease worsening in multiple sclerosis. Multiple sclerosis (Houndmills, Basingstoke, England). 2022;28(12):1859-70.

23. Burman J, Raininko R, Blennow K, Zetterberg H, Axelsson M, Malmestrom C. YKL-40 is a CSF biomarker of intrathecal inflammation in secondary progressive multiple sclerosis. Journal of neuroimmunology. 2016;292(JA2, 8109498, hso):52-7.

24. Burman J, Zetterberg H, Fransson M, Loskog AS, Raininko R, Fagius J. Assessing tissue damage in multiple sclerosis: a biomarker approach. Acta neurologica Scandinavica. 2014;130(2):81-9.

25. Bystrom S, Ayoglu B, Haggmark A, Mitsios N, Hong M-G, Drobin K, et al. Affinity proteomic profiling of plasma, cerebrospinal fluid, and brain tissue within multiple sclerosis. Journal of proteome research. 2014;13(11):4607-19.

26. Casanova B, Castillo J, Quintanilla-Bordas C, Sanz MT, Fernandez-Velasco JI, Alcala C, et al. Oligoclonal M bands unveil occult inflammation in multiple sclerosis. Multiple sclerosis and related disorders. 2022;68(101580247):104118.

27. Chatterjee M, Koel-Simmelink MJ, Verberk IM, Killestein J, Vrenken H, Enzinger C, et al. Contactin-1 and contactin-2 in cerebrospinal fluid as potential biomarkers for axonal domain dysfunction in multiple sclerosis. Multiple sclerosis journal - experimental, translational and clinical. 2018;4(4):2055217318819535.

28. Checa A, Khademi M, Sar DG, Haeggstrom JZ, Lundberg JO, Piehl F, et al. Hexosylceramides as intrathecal markers of worsening disability in multiple sclerosis. Multiple sclerosis (Houndmills, Basingstoke, England). 2015;21(10):1271-9.

29. Collongues N, Kuhle J, Tsagkas C, Lamy J, Meyer N, Barro C, et al. Biomarkers of treatment response in patients with progressive multiple sclerosis treated with high-dose pharmaceutical-grade biotin (MD1003). Brain and behavior. 2021;11(2):e01998.

30. Comabella M, Sastre-Garriga J, Carbonell-Mirabent P, Fissolo N, Tur C, Malhotra S, et al. Serum neurofilament light chain levels predict long-term disability progression in patients with progressive multiple sclerosis. Journal of neurology, neurosurgery, and psychiatry. 2022(2985191r, jbb).

31. Cristofanilli M, Gratch D, Pagano B, McDermott K, Huang J, Jian J, et al. Transglutaminase-6 is an autoantigen in progressive multiple sclerosis and is upregulated in reactive astrocytes. Multiple sclerosis (Houndmills, Basingstoke, England). 2017;23(13):1707-15.

32. Cubas-Nunez L, Gil-Perotin S, Castillo-Villalba J, Lopez V, Solis Tarazona L, Gasque-Rubio R, et al. Potential Role of CHI3L1+ Astrocytes in Progression in MS. Neurology(R) neuroimmunology & neuroinflammation. 2021;8(3).

33. Damasceno A, Dias-Carneiro RPC, Moraes AS, Boldrini VO, Quintiliano RPS, da Silva VAdPG, et al. Clinical and MRI correlates of CSF neurofilament light chain levels in relapsing and progressive MS. Multiple sclerosis and related disorders. 2019;30(101580247):149-53.

34. Dietmann A-S, Kruse N, Stork L, Gloth M, Bruck W, Metz I. Neurofilament light chains in serum as biomarkers of axonal damage in early MS lesions: a histological-serological correlative study. Journal of neurology. 2022(jb7, 0423161).

35. Disanto G, Barro C, Benkert P, Naegelin Y, Schadelin S, Giardiello A, et al. Serum Neurofilament light: A biomarker of neuronal damage in multiple sclerosis. Annals of neurology. 2017;81(6):857-70.

36. Disanto G, Ripellino P, Riccitelli GC, Sacco R, Scotti B, Fucili A, et al. De-escalating rituximab dose results in stability of clinical, radiological, and serum neurofilament levels in multiple sclerosis. Multiple sclerosis (Houndmills, Basingstoke, England). 2021;27(8):1230-9.

37. Edwards KR, Kamath A, Button J, Kamath V, Mendoza JP, Zhu B, et al. A pharmacokinetic and biomarker study of delayed-release dimethyl fumarate in subjects with secondary progressive multiple sclerosis: evaluation of cerebrospinal fluid penetration and the effects on exploratory biomarkers. Multiple sclerosis and related disorders. 2021;51(101580247):102861.

38. Eikelenboom MJ, Uitdehaag BMJ, Petzold A. Blood and CSF Biomarker Dynamics in Multiple Sclerosis: Implications for Data Interpretation. Multiple sclerosis international. 2011;2011(101566861):823176.

39. Elkjaer ML, Nawrocki A, Kacprowski T, Lassen P, Simonsen AH, Marignier R, et al. CSF proteome in multiple sclerosis subtypes related to brain lesion transcriptomes. Scientific reports. 2021;11(1):4132.

40. Ferraro D, Guicciardi C, De Biasi S, Pinti M, Bedin R, Camera V, et al. Plasma neurofilaments correlate with disability in progressive multiple sclerosis patients. Acta neurologica Scandinavica. 2020;141(1):16-21.

41. Fialova L, Bartos A, Svarcova J, Malbohan I. Increased intrathecal high-avidity anti-tau antibodies in patients with multiple sclerosis. PloS one. 2011;6(11):e27476.

42. Filippatou AG, Moniruzzaman M, Sotirchos ES, Fitzgerald KC, Kalaitzidis G, Lambe J, et al. Serum ceramide levels are altered in multiple sclerosis. Multiple sclerosis (Houndmills, Basingstoke, England). 2021;27(10):1506-19.

43. Gaetani L, Hoglund K, Parnetti L, Pujol-Calderon F, Becker B, Eusebi P, et al. A new enzyme-linked immunosorbent assay for neurofilament light in cerebrospinal fluid: analytical validation and clinical evaluation. Alzheimer's research & therapy. 2018;10(1):8.

44. Gauthier A, Viel S, Perret M, Brocard G, Casey R, Lombard C, et al. Comparison of SimoaTM and EllaTM to assess serum neurofilament-light chain in multiple sclerosis. Annals of clinical and translational neurology. 2021;8(5):1141-50.

45. Gil-Perotin S, Castillo-Villalba J, Cubas-Nunez L, Gasque R, Hervas D, Gomez-Mateu J, et al. Combined Cerebrospinal Fluid Neurofilament Light Chain Protein and Chitinase-3 Like-1 Levels in Defining Disease Course and Prognosis in Multiple Sclerosis. Frontiers in neurology. 2019;10(101546899):1008.

46. Gjelstrup MC, Stilund M, Petersen T, Moller HJ, Petersen EL, Christensen T. Subsets of activated monocytes and markers of inflammation in incipient and progressed multiple sclerosis. Immunology and cell biology. 2018;96(2):160-74.

47. Gnanapavan S, Yousaf N, Heywood W, Grant D, Mills K, Chernajovsky Y, et al. Growth associated protein (GAP-43): cloning and the development of a sensitive ELISA for neurological disorders. Journal of neuroimmunology. 2014;276(1-2):18-23.

48. Greer JM, Trifilieff E, Pender MP. Correlation Between Anti-Myelin Proteolipid Protein (PLP) Antibodies and Disease Severity in Multiple Sclerosis Patients With PLP Response-Permissive HLA Types. Frontiers in immunology. 2020;11(101560960):1891.

49. Gresle MM, Liu Y, Dagley LF, Haartsen J, Pearson F, Purcell AW, et al. Serum phosphorylated neurofilament-heavy chain levels in multiple sclerosis patients. Journal of neurology, neurosurgery, and psychiatry. 2014;85(11):1209-13.

50. Haggmark A, Bystrom S, Ayoglu B, Qundos U, Uhlen M, Khademi M, et al. Antibody-based profiling of cerebrospinal fluid within multiple sclerosis. Proteomics. 2013;13(15):2256-67.

51. Haghighi S, Lekman A, Nilsson S, Blomqvist M, Andersen O. Myelin glycosphingolipid immunoreactivity and CSF levels in multiple sclerosis. Acta neurologica Scandinavica. 2012;125(1):64-70.

52. Hares K, Kemp K, Loveless S, Rice CM, Scolding N, Tallantyre E, et al. KIF5A and the contribution of susceptibility genotypes as a predictive biomarker for multiple sclerosis. Journal of neurology. 2021;268(6):2175-84.

53. Harp C, Thanei G-A, Jia X, Kuhle J, Leppert D, Schaedelin S, et al. Development of an age-adjusted model for blood neurofilament light chain. Annals of clinical and translational neurology. 2022;9(4):444-53.

54. Harris VK, Diamanduros A, Good P, Zakin E, Chalivendra V, Sadiq SA. Bri2-23 is a potential cerebrospinal fluid biomarker in multiple sclerosis. Neurobiology of disease. 2010;40(1):331-9.

55. Hebb ALO, Bhan V, Wishart AD, Moore CS, Robertson GS. Human kallikrein 6 cerebrospinal levels are elevated in multiple sclerosis. Current drug discovery technologies. 2010;7(2):137-40.

56. Hendricks R, Baker D, Brumm J, Davancaze T, Harp C, Herman A, et al. Establishment of neurofilament light chain Simoa assay in cerebrospinal fluid and blood. Bioanalysis. 2019;11(15):1405-18.

57. Hjaeresen S, Sejbaek T, Axelsson M, Vinslov-Jensen H, Mortensen SK, Pihl-Jensen G, et al. The levels of the serine protease HTRA1 in cerebrospinal fluid correlate with progression and disability in multiple sclerosis. Journal of neurology. 2021;268(9):3316-24.

58. Hogel H, Rissanen E, Barro C, Matilainen M, Nylund M, Kuhle J, et al. Serum glial fibrillary acidic protein correlates with multiple sclerosis disease severity. Multiple sclerosis (Houndmills, Basingstoke, England). 2020;26(2):210-9.

59. Ineichen BV, Moridi T, Ewing E, Ouellette R, Manouchehrinia A, Stawiarz L, et al. Neurofilament light chain as a marker for cortical atrophy in multiple sclerosis without radiological signs of disease activity. Journal of internal medicine. 2021;290(2):473-6.

60. Ioannides ZA, Csurhes PA, Swayne A, Foubert P, Aftab BT, Pender MP. Correlations between macrophage/microglial activation marker sTREM-2 and measures of T-cell activation, neuroaxonal damage and disease severity in multiple sclerosis. Multiple sclerosis journal - experimental, translational and clinical. 2021;7(2):20552173211019772.

61. Jakimovski D, Bergsland N, Dwyer MG, Ramasamy DP, Ramanathan M, Weinstock-Guttman B, et al. Serum Neurofilament Light Chain Levels are Associated with Lower Thalamic Perfusion in Multiple Sclerosis. Diagnostics (Basel, Switzerland). 2020;10(9).

62. Jakimovski D, Gibney BL, Marr K, Ramasamy DP, Dwyer MG, Bergsland N, et al. Lower cerebral arterial blood flow is associated with greater serum neurofilament light chain levels in multiple sclerosis patients. European journal of neurology. 2022;29(8):2299-308.

63. Jakimovski D, Kuhle J, Ramanathan M, Barro C, Tomic D, Hagemeier J, et al. Serum neurofilament light chain levels associations with gray matter pathology: a 5-year longitudinal study. Annals of clinical and translational neurology. 2019;6(9):1757-70.

64. Jakimovski D, Zivadinov R, Dwyer MG, Bergsland N, Ramasamy DP, Browne RW, et al. High density lipoprotein cholesterol and apolipoprotein A-I are associated with greater cerebral perfusion in multiple sclerosis. Journal of the neurological sciences. 2020;418(jbj, 0375403):117120.

65. Jakimovski D, Zivadinov R, Ramanthan M, Hagemeier J, Weinstock-Guttman B, Tomic D, et al. Serum neurofilament light chain level associations with clinical and cognitive performance in multiple sclerosis: A longitudinal retrospective 5-year study. Multiple sclerosis (Houndmills, Basingstoke, England). 2020;26(13):1670-81.

66. Jaworski J, Psujek M, Janczarek M, Szczerbo-Trojanowska M, Bartosik-Psujek H. Total-tau in cerebrospinal fluid of patients with multiple sclerosis decreases in secondary progressive stage of disease and reflects degree of brain atrophy. Upsala journal of medical sciences. 2012;117(3):284-92.

67. Jia Y, Wu T, Jelinek CA, Bielekova B, Chang L, Newsome S, et al. Development of protein biomarkers in cerebrospinal fluid for secondary progressive multiple sclerosis using selected reaction monitoring mass spectrometry (SRM-MS). Clinical proteomics. 2012;9(1):9.

68. Kalatha T, Arnaoutoglou M, Koukoulidis T, Hatzifilippou E, Bouras E, Baloyannis S, et al. Does cognitive dysfunction correlate with neurofilament light polypeptide levels in the CSF of patients with multiple sclerosis? The Journal of international medical research. 2019;47(5):2187-98.

69. Kessler C, Ruschil C, Abdelhak A, Wilke C, Maleska A, Kuhle J, et al. Serum Neurofilament Light Chain and Glial Fibrillary Acidic Protein as Biomarkers in Primary Progressive Multiple Sclerosis and Hereditary Spastic Paraplegia Type 4. International journal of molecular sciences. 2022;23(21).

70. Kirk PDW, Witkover A, Courtney A, Lewin AM, Wait R, Stumpf MPH, et al. Plasma proteome analysis in HTLV-1-associated myelopathy/tropical spastic paraparesis. Retrovirology. 2011;8(101216893):81.

71. Klawiter EC, Piccio L, Lyons J-A, Mikesell R, O'Connor KC, Cross AH. Elevated intrathecal myelin oligodendrocyte glycoprotein antibodies in multiple sclerosis. Archives of neurology. 2010;67(9):1102-8.

72. Koch MW, George S, Wall W, Wee Yong V, Metz LM. Serum NSE level and disability progression in multiple sclerosis. Journal of the neurological sciences. 2015;350(1-2):46-50.

73. Kosehasanogullari G, Ozakbas S, Idiman E. Tau protein levels in the cerebrospinal fluid of the patients with multiple sclerosis in an attack period: Low levels of tau protein may have significance, too. Clinical neurology and neurosurgery. 2015;136(df4, 7502039):107-9.

74. Kuhle J, Leppert D, Petzold A, Regeniter A, Schindler C, Mehling M, et al. Neurofilament heavy chain in CSF correlates with relapses and disability in multiple sclerosis. Neurology. 2011;76(14):1206-13.

75. Kuhle J, Plattner K, Bestwick JP, Lindberg RL, Ramagopalan SV, Norgren N, et al. A comparative study of CSF neurofilament light and heavy chain protein in MS. Multiple sclerosis (Houndmills, Basingstoke, England). 2013;19(12):1597-603.

76. Lamancova P, Urban P, Maslankova J, Rabajdova M, Marekova M. Correlation of selected serum protein levels with the degree of disability and NEDA-3 status in multiple sclerosis phenotypes. European review for medical and pharmacological sciences. 2022;26(11):3933-41.

77. Leppert D, Kropshofer H, Haring DA, Dahlke F, Patil A, Meinert R, et al. Blood Neurofilament Light in Progressive Multiple Sclerosis: Post Hoc Analysis of 2 Randomized Controlled Trials. Neurology. 2022;98(21):e2120-e31.

78. Leurs CE, Lopes Pinheiro MA, Wierts L, den Hoedt S, Mulder MT, Eijlers AJC, et al. Acid sphingomyelinase: No potential as a biomarker for multiple sclerosis. Multiple sclerosis and related disorders. 2019;28(101580247):44-9.

79. Madeddu R, Farace C, Tolu P, Solinas G, Asara Y, Sotgiu MA, et al. Cytoskeletal proteins in the cerebrospinal fluid as biomarker of multiple sclerosis. Neurological sciences : official journal of the Italian Neurological Society and of the Italian Society of Clinical Neurophysiology. 2013;34(2):181-6.

80. Maggi P, Kuhle J, Schadelin S, van der Meer F, Weigel M, Galbusera R, et al. Chronic White Matter Inflammation and Serum Neurofilament Levels in Multiple Sclerosis. Neurology. 2021;97(6):e543-e53.

81. Mandoj C, Renna R, Plantone D, Sperduti I, Cigliana G, Conti L, et al. Anti-annexin antibodies, cholesterol levels and disability in multiple sclerosis. Neuroscience letters. 2015;606(n7n, 7600130):156-60.

82. Mane-Martinez MA, Olsson B, Bau L, Matas E, Cobo-Calvo A, Andreasson U, et al. Glial and neuronal markers in cerebrospinal fluid in different types of multiple sclerosis. Journal of neuroimmunology. 2016;299(JA2, 8109498, hso):112-7.

83. Manouchehrinia A, Stridh P, Khademi M, Leppert D, Barro C, Michalak Z, et al. Plasma neurofilament light levels are associated with risk of disability in multiple sclerosis. Neurology. 2020;94(23):e2457-e67.

84. Mariottini A, Marchi L, Innocenti C, Di Cristinzi M, Pasca M, Filippini S, et al. Intermediate-Intensity Autologous Hematopoietic Stem Cell Transplantation Reduces Serum Neurofilament Light Chains and Brain Atrophy in Aggressive Multiple Sclerosis. Frontiers in neurology. 2022;13(101546899):820256.

85. Martin NA, Nawrocki A, Molnar V, Elkjaer ML, Thygesen EK, Palkovits M, et al. Orthologous proteins of experimental de- and remyelination are differentially regulated in the CSF proteome of multiple sclerosis subtypes. PloS one. 2018;13(8):e0202530.

86. McComb M, Parambi R, Browne RW, Bodziak ML, Jakimovski D, Bergsland N, et al. Apolipoproteins AI and E are associated with neuroaxonal injury to gray matter in multiple sclerosis. Multiple sclerosis and related disorders. 2020;45(101580247):102389.

87. Melief J, de Wit SJ, van Eden CG, Teunissen C, Hamann J, Uitdehaag BM, et al. HPA axis activity in multiple sclerosis correlates with disease severity, lesion type and gene expression in normal-appearing white matter. Acta neuropathologica. 2013;126(2):237-49.

88. Meyers L, Groover CJ, Douglas J, Lee S, Brand D, Levin MC, et al. A role for Apolipoprotein A-I in the pathogenesis of multiple sclerosis. Journal of neuroimmunology. 2014;277(1-2):176-85.

89. Miyachi Y, Fujii T, Yamasaki R, Tsuchimoto D, Iinuma K, Sakoda A, et al. Serum Anti-oligodendrocyte Autoantibodies in Patients With Multiple Sclerosis Detected by a Tissue-Based Immunofluorescence Assay. Frontiers in neurology. 2021;12(101546899):681980.

90. Murgia F, Lorefice L, Poddighe S, Fenu G, Secci MA, Marrosu MG, et al. Multi-Platform Characterization of Cerebrospinal Fluid and Serum Metabolome of Patients Affected by Relapsing-Remitting and Primary Progressive Multiple Sclerosis. Journal of clinical medicine. 2020;9(3).

91. Nova A, Fazia T, Beecham A, Saddi V, Piras M, McCauley JL, et al. Plasma Protein Levels Analysis in Multiple Sclerosis Sardinian Families Identified C9 and CYP24A1 as Candidate Biomarkers. Life (Basel, Switzerland). 2022;12(2).

92. Novakova L, Axelsson M, Malmestrom C, Imberg H, Elias O, Zetterberg H, et al. Searching for neurodegeneration in multiple sclerosis at clinical onset: Diagnostic value of biomarkers. PloS one. 2018;13(4):e0194828.

93. Novakova L, Singh AK, Axelsson M, Stahlman M, Adiels M, Malmestrom C, et al. Sulfatide isoform pattern in cerebrospinal fluid discriminates progressive MS from relapsing-remitting MS. Journal of neurochemistry. 2018;146(3):322-32.

94. Novakova L, Zetterberg H, Sundstrom P, Axelsson M, Khademi M, Gunnarsson M, et al. Monitoring disease activity in multiple sclerosis using serum neurofilament light protein. Neurology. 2017;89(22):2230-7.

95. Ottervald J, Franzen B, Nilsson K, Andersson LI, Khademi M, Eriksson B, et al. Multiple sclerosis: Identification and clinical evaluation of novel CSF biomarkers. Journal of proteomics. 2010;73(6):1117-32.

96. Pauwels A, Van Schependom J, Devolder L, Van Remoortel A, Nagels G, Bjerke M, et al. Plasma glial fibrillary acidic protein and neurofilament light chain in relation to disability worsening in multiple sclerosis. Multiple sclerosis (Houndmills, Basingstoke, England). 2022;28(11):1685-96.

97. Pawlitzki M, Schreiber S, Bittner D, Kreipe J, Leypoldt F, Rupprecht K, et al. CSF Neurofilament Light Chain Levels in Primary Progressive MS: Signs of Axonal Neurodegeneration. Frontiers in neurology. 2018;9(101546899):1037.

98. Pawlitzki M, Sweeney-Reed CM, Bittner D, Lux A, Vielhaber S, Schreiber S, et al. CSF-Progranulin and Neurofilament Light Chain Levels in Patients With Radiologically Isolated Syndrome-Sign of Inflammation. Frontiers in neurology. 2018;9(101546899):1075.

99. Petitfour J, Ayrignac X, Ginestet N, Prin P, Carra-Dalliere C, Hirtz C, et al. CSF beta-amyloid is not a prognostic marker in multiple sclerosis patients. Multiple sclerosis and related disorders. 2022;68(101580247):104096.

100. Petzold A. The prognostic value of CSF neurofilaments in multiple sclerosis at 15-year follow-up. Journal of neurology, neurosurgery, and psychiatry. 2015;86(12):1388-90.

101. Petzold A, Mondria T, Kuhle J, Rocca MA, Cornelissen J, te Boekhorst P, et al. Evidence for acute neurotoxicity after chemotherapy. Annals of neurology. 2010;68(6):806-15.

102. Piehl F, Kockum I, Khademi M, Blennow K, Lycke J, Zetterberg H, et al. Plasma neurofilament light chain levels in patients with MS switching from injectable therapies to fingolimod. Multiple sclerosis (Houndmills, Basingstoke, England). 2018;24(8):1046-54.

103. Pietroboni AM, Carandini T, Colombi A, Mercurio M, Ghezzi L, Giulietti G, et al. Amyloid PET as a marker of normal-appearing white matter early damage in multiple sclerosis: correlation with CSF beta-amyloid levels and brain volumes. European journal of nuclear medicine and molecular imaging. 2019;46(2):280-7.

104. Podbielska M, Dasgupta S, Levery SB, Tourtellotte WW, Annuk H, Moran AP, et al. Novel myelin penta- and hexa-acetyl-galactosyl-ceramides: structural characterization and immunoreactivity in cerebrospinal fluid. Journal of lipid research. 2010;51(6):1394-406.

105. Proschmann U, Inojosa H, Akgun K, Ziemssen T. Natalizumab Pharmacokinetics and -Dynamics and Serum Neurofilament in Patients With Multiple Sclerosis. Frontiers in neurology. 2021;12(101546899):650530.

106. Puentes F, Benkert P, Amor S, Kuhle J, Giovannoni G. Antibodies to neurofilament light as potential biomarkers in multiple sclerosis. BMJ neurology open. 2021;3(2):e000192.

107. Reyes S, Smets I, Holden D, Carrillo-Loza K, Christmas T, Bianchi L, et al. CSF neurofilament light chain testing as an aid to determine treatment strategies in MS. Neurology(R) neuroimmunology & neuroinflammation. 2020;7(6).

108. Rise HH, Brune S, Chien C, Berge T, Bos SD, Andorra M, et al. Brain disconnectome mapping derived from white matter lesions and serum neurofilament light levels in multiple sclerosis: A longitudinal multicenter study. NeuroImage Clinical. 2022;35(101597070):103099.

109. Romme Christensen J, Bornsen L, Khademi M, Olsson T, Jensen PE, Sorensen PS, et al. CSF inflammation and axonal damage are increased and correlate in progressive multiple sclerosis. Multiple sclerosis (Houndmills, Basingstoke, England). 2013;19(7):877-84.

110. Romme Christensen J, Komori M, von Essen MR, Ratzer R, Bornsen L, Bielekova B, et al. CSF inflammatory biomarkers responsive to treatment in progressive multiple sclerosis capture residual inflammation associated with axonal damage. Multiple sclerosis (Houndmills, Basingstoke, England). 2019;25(7):937-46.

111. Rosso M, Healy BC, Saxena S, Paul A, Bjornevik K, Kuhle J, et al. MRI Lesion State Modulates the Relationship Between Serum Neurofilament Light and Age in Multiple Sclerosis. Journal of neuroimaging : official journal of the American Society of Neuroimaging. 2021;31(2):388-93.

112. Rot U, Sandelius A, Emersic A, Zetterberg H, Blennow K. Cerebrospinal fluid GAP-43 in early multiple sclerosis. Multiple sclerosis journal - experimental, translational and clinical. 2018;4(3):2055217318792931.

113. Sadaba MC, Rothhammer V, Munoz U, Sebal C, Escudero E, Kivisakk P, et al. Serum antibodies to phosphatidylcholine in MS. Neurology(R) neuroimmunology & neuroinflammation. 2020;7(4).

114. Salzer J, Svenningsson A, Sundstrom P. Neurofilament light as a prognostic marker in multiple sclerosis. Multiple sclerosis (Houndmills, Basingstoke, England). 2010;16(3):287-92.

115. Sandelius A, Sandgren S, Axelsson M, Malmestrom C, Novakova L, Kostanjevecki V, et al. Cerebrospinal fluid growth-associated protein 43 in multiple sclerosis. Scientific reports. 2019;9(1):17309.

116. Saraste M, Bezukladova S, Matilainen M, Tuisku J, Rissanen E, Sucksdorff M, et al. High serum neurofilament associates with diffuse white matter damage in MS. Neurology(R) neuroimmunology & neuroinflammation. 2021;8(1).

117. Schneider R, Bellenberg B, Gisevius B, Hirschberg S, Sankowski R, Prinz M, et al. Chitinase 3-like 1 and neurofilament light chain in CSF and CNS atrophy in MS. Neurology(R) neuroimmunology & neuroinflammation. 2021;8(1).

118. Sellebjerg F, Bornsen L, Ammitzboll C, Nielsen JE, Vinther-Jensen T, Hjermind LE, et al. Defining active progressive multiple sclerosis. Multiple sclerosis (Houndmills, Basingstoke, England). 2017;23(13):1727-35.

119. Senanayake VK, Jin W, Mochizuki A, Chitou B, Goodenowe DB. Metabolic dysfunctions in multiple sclerosis: implications as to causation, early detection, and treatment, a case control study. BMC neurology. 2015;15(100968555):154.

120. Shi Y, Ding Y, Li G, Wang L, Osman RA, Sun J, et al. Discovery of Novel Biomarkers for Diagnosing and Predicting the Progression of Multiple Sclerosis Using TMT-Based Quantitative Proteomics. Frontiers in immunology. 2021;12(101560960):700031.

121. Siddiqui K, Browne RW, Benedict RHB, Jakimovski D, Weinstock-Guttman B, Zivadinov R, et al. Cholesterol pathway biomarkers are associated with neuropsychological measures in multiple sclerosis. Multiple sclerosis and related disorders. 2022;69(101580247):104374.

122. Smets I, Holden D, Bianchi L, Ammoscato F, Allen-Philbey K, Baker D, et al. Factors contributing to CSF NfL reduction over time in those starting treatment for multiple sclerosis: An observational study. Multiple sclerosis and related disorders. 2022;57(101580247):103409.

123. Sotirchos ES, Fitzgerald KC, Singh CM, Smith MD, Reyes-Mantilla M, Hersh CM, et al. Associations of sNfL with clinico-radiological measures in a large MS population. Annals of clinical and translational neurology. 2022(101623278).

124. Stich O, Perera S, Berger B, Jarius S, Wildemann B, Baumgartner A, et al. Prevalence of neurofascin-155 antibodies in patients with multiple sclerosis. Journal of the neurological sciences. 2016;364(jbj, 0375403):29-32.

125. Stilund M, Gjelstrup MC, Petersen T, Moller HJ, Rasmussen PV, Christensen T. Biomarkers of inflammation and axonal degeneration/damage in patients with newly diagnosed multiple sclerosis: contributions of the soluble CD163 CSF/serum ratio to a biomarker panel. PloS one. 2015;10(4):e0119681.

126. Stoop MP, Singh V, Dekker LJ, Titulaer MK, Stingl C, Burgers PC, et al. Proteomics comparison of cerebrospinal fluid of relapsing remitting and primary progressive multiple sclerosis. PloS one. 2010;5(8):e12442.

127. Szalardy L, Zadori D, Simu M, Bencsik K, Vecsei L, Klivenyi P. Evaluating biomarkers of neuronal degeneration and neuroinflammation in CSF of patients with multiple sclerosis-osteopontin as a potential marker of clinical severity. Journal of the neurological sciences. 2013;331(1-2):38-42.

128. Talbot J, Hojsgaard Chow H, Mahler M, Buhelt S, Holm Hansen R, Lundell H, et al. Relationship between cerebrospinal fluid biomarkers of inflammation and tissue damage in primary progressive multiple sclerosis. Multiple sclerosis and related disorders. 2022;68(101580247):104209.

129. Tavazzi E, Bergsland N, Kuhle J, Jakimovski D, Ramanathan M, Maceski AM, et al. A multimodal approach to assess the validity of atrophied T2-lesion volume as an MRI marker of disease progression in multiple sclerosis. Journal of neurology. 2020;267(3):802-11.

130. Tavazzi E, Jakimovski D, Kuhle J, Hagemeier J, Ozel O, Ramanathan M, et al. Serum neurofilament light chain and optical coherence tomography measures in MS: A longitudinal study. Neurology(R) neuroimmunology & neuroinflammation. 2020;7(4).

131. Tenorio-Laranga J, Peltonen I, Keskitalo S, Duran-Torres G, Natarajan R, Mannisto PT, et al. Alteration of prolyl oligopeptidase and activated alpha-2-macroglobulin in multiple sclerosis subtypes and in the clinically isolated syndrome. Biochemical pharmacology. 2013;85(12):1783-94.

132. Tewarie P, Teunissen CE, Dijkstra CD, Heijnen DAM, Vogt M, Balk L, et al. Cerebrospinal fluid anti-whole myelin antibodies are not correlated to magnetic resonance imaging activity in multiple sclerosis. Journal of neuroimmunology. 2012;251(1-2):103-6.

133. Thebault S, Abdoli M, Fereshtehnejad S-M, Tessier D, Tabard-Cossa V, Freedman MS. Serum neurofilament light chain predicts long term clinical outcomes in multiple sclerosis. Scientific reports. 2020;10(1):10381.

134. Thebault S, R Tessier D, Lee H, Bowman M, Bar-Or A, Arnold DL, et al. High serum neurofilament light chain normalizes after hematopoietic stem cell transplantation for MS. Neurology(R) neuroimmunology & neuroinflammation. 2019;6(5):e598.

135. Thebault S, Reaume M, Marrie RA, Marriott JJ, Furlan R, Laroni A, et al. High or increasing serum NfL is predictive of impending multiple sclerosis relapses. Multiple sclerosis and related disorders. 2022;59(101580247):103535.

136. Tottenham I, Koch M, Camara-Lemarroy C. Serum HGF and APN2 are associated with disability worsening in SPMS. Journal of neuroimmunology. 2022;364(JA2, 8109498, hso):577803.

137. Trentini A, Comabella M, Tintore M, Koel-Simmelink MJA, Killestein J, Roos B, et al. N-acetylaspartate and neurofilaments as biomarkers of axonal damage in patients with progressive forms of multiple sclerosis. Journal of neurology. 2014;261(12):2338-43.

138. Tsagkas C, Naegelin Y, Amann M, Papadopoulou A, Barro C, Chakravarty MM, et al. Central nervous system atrophy predicts future dynamics of disability progression in a real-world multiple sclerosis cohort. European journal of neurology. 2021;28(12):4153-66.

139. Tsagkas C, Parmar K, Pezold S, Barro C, Chakravarty MM, Gaetano L, et al. Classification of multiple sclerosis based on patterns of CNS regional atrophy covariance. Human brain mapping. 2021;42(8):2399-415.

140. Uzunkopru C, Yuceyar N, Yilmaz SG, Afrashi F, Ekmekci O, Taskiran D. Retinal Nerve Fiber Layer Thickness Correlates with Serum and Cerebrospinal Fluid Neurofilament Levels and is Associated with Current Disability in Multiple Sclerosis. Noro psikiyatri arsivi. 2021;58(1):34-40.

141. van den Bosch A, Fransen N, Mason M, Rozemuller AJ, Teunissen C, Smolders J, et al. Neurofilament Light Chain Levels in Multiple Sclerosis Correlate With Lesions Containing Foamy Macrophages and With Acute Axonal Damage. Neurology(R) neuroimmunology & neuroinflammation. 2022;9(3).

142. Van Hijfte L, Loret G, Bachmann H, Reynders T, Breuls M, Deschepper E, et al. Lifestyle factors in multiple sclerosis disability progression and silent brain damage: A cross-sectional study. Multiple sclerosis and related disorders. 2022;65(101580247):104016.

143. Verberk IMW, Koel-Simmelink M, Twaalfhoven H, Vrenken H, Korth C, Killestein J, et al. Ultrasensitive immunoassay allows measurement of serum neurofilament heavy in multiple sclerosis. Multiple sclerosis and related disorders. 2021;50(101580247):102840.

144. Virgilio E, Vecchio D, Crespi I, Serino R, Cantello R, Dianzani U, et al. Cerebrospinal Tau levels as a predictor of early disability in multiple sclerosis. Multiple sclerosis and related disorders. 2021;56(101580247):103231.

145. Vuletic S, Kennedy H, Albers JJ, Killestein J, Vrenken H, Lutjohann D, et al. Cerebrospinal fluid apolipoprotein E and phospholipid transfer protein activity are reduced in multiple sclerosis; relationships with the brain MRI and CSF lipid variables. Multiple sclerosis and related disorders. 2014;3(4):533-41.

146. Watanabe M, Nakamura Y, Michalak Z, Isobe N, Barro C, Leppert D, et al. Serum GFAP and neurofilament light as biomarkers of disease activity and disability in NMOSD. Neurology. 2019;93(13):e1299-e311.

147. Yik JT, Becquart P, Gill J, Petkau J, Traboulsee A, Carruthers R, et al. Serum neurofilament light chain correlates with myelin and axonal magnetic resonance imaging markers in multiple sclerosis. Multiple sclerosis and related disorders. 2022;57(101580247):103366.
